# Modeling the Treatment of Glioblastoma Multiforme and Cancer Stem Cells with Ordinary Differential Equations

**DOI:** 10.1155/2016/1239861

**Published:** 2016-01-18

**Authors:** Kristen Abernathy, Jeremy Burke

**Affiliations:** ^1^Department of Mathematics, Winthrop University, Rock Hill, SC 29733, USA; ^2^Department of Mathematics, Vassar College, Poughkeepsie, NY 12604, USA

## Abstract

Despite improvements in cancer therapy and treatments, tumor recurrence is a common event in cancer patients. One explanation of recurrence is that cancer therapy focuses on treatment of tumor cells and does not eradicate cancer stem cells (CSCs). CSCs are postulated to behave similar to normal stem cells in that their role is to maintain homeostasis. That is, when the population of tumor cells is reduced or depleted by treatment, CSCs will repopulate the tumor, causing recurrence. In this paper, we study the application of the CSC Hypothesis to the treatment of glioblastoma multiforme by immunotherapy. We extend the work of Kogan et al. (2008) to incorporate the dynamics of CSCs, prove the existence of a recurrence state, and provide an analysis of possible cancerous states and their dependence on treatment levels.

## 1. Introduction

Dynamical systems continue to play an important role in understanding cancer dynamics [1–4]. A recent development in cancer dynamics is the cancer stem cell hypothesis. The cancer stem cell hypothesis states that malignant tumors are initiated and maintained by a population of tumor cells that share similar biologic properties to normal adult stem cells [5]. With evidence mounting in support of the cancer stem cell hypothesis, recent work has been devoted to the inclusion of cancer stem cells (CSCs) in current cancer models [6–9].

Cancer stem cells are a specialized type of cancer cell that are believed to be responsible for populating tumors. CSCs have a very small population in comparison to normal cancer cells because of their specialized function. While tumor cells are only able to undergo a limited number of divisions, CSCs are able to repopulate a depleted tumor, even if there are only a few CSCs left [10]. Once the number of CSCs begins to drop, usually due to treatment, they cease creating cancer cells and focus on repopulating themselves. The small population of CSCs is hard to detect and therefore treatment is often stopped before all CSCs have been eradicated, which leads to recurrence of cancer [11]. It is clear that, for treatment to be effective, we must focus our efforts on eliminating both tumor cells and CSCs.

The cancer stem cell hypothesis has been biologically verified for many solid tumors, including brain cancer [12]. Glioblastoma is a type of brain tumor which forms in the cerebral hemisphere of the brain, often in the frontal and temporal lobe. These types of tumors are highly malignant, forming from normal brain cells, astrocytes, or star-shaped glial cells which support nerve cells. These cells can grow rapidly due to ample amounts of blood available in the brain. Immunotherapy is a cancer treatment which stimulates the immune system to work harder to attack cancer cells. The therapy uses additive components, such as man-made proteins, vaccines, or white blood cells, to further attack cancer cells. Immunotherapy is essential to treating multiforme glioblastomas because of their sensitive location, the brain, which is too delicate to be treated by chemotherapy or surgery [10, 13].

## 2. Presentation of the Model

In this paper, we extend the previous work of Kogan et al. [13] to include cancer stem cells in modeling the treatment of glioblastoma multiforme with immunotherapy. We present an abstract model that can be adapted to fit various biological assumptions. We analyze the stability of the model both with and without treatment and derive sufficient conditions on treatment to ensure a globally asymptotically stable cure state. We conclude with an example illustrating the transition from coexistence of cancer cells to eradication of cancer cells with various treatment levels. Much of this model is based on experimental results obtained by Kruse et al. [14].

The following system models the dynamics of tumor cells (*T*), cancer stem cells (*S*), alloreactive cytotoxic-T-lymphocytes (*C*), TGF-*β* (*F*_*β*_), IFN-*γ* (*F*_*γ*_), and major histocompatibility complex classes I and II (*M*_I_ and *M*_II_, resp.):

(1) T′=αT,S+R1TT−fTFβgTMIhTTCT,S′=R2SS−αT,S−fSFβgSMIhSSCS,C′=fCT+S·MIIgCFβ−μCC+Nt,Fβ′=fβT+S−μβFβ,Fγ′=fγC−μγFγ,MI′=fMIFγ−μMIMI,MII′=fMIIFβgMIIFγ−μMIIMII.

To understand the formation of the system above, we discuss the biological interpretations of each equation in the model:

(2) $$\begin{array}{c} T^{\prime }=\alpha \left(\;T,S\;\right)+R_{1}\left(\;T\;\right)T-f_{T}\left(\;F_{\beta }\;\right)g_{T}\left(\;M_{I}\;\right)h_{T}\left(\;T\;\right)CT. \end{array}$$

The first term on the right hand side (RHS) of the equation represents differentiated tumor cells produced by the CSCs without immune intervention, where *α*(*T*, *S*) is the rate at which CSCs produce tumor cells and *T* is the number of nonstem tumor cells (TCs) currently present. The second term stands for normal tumor growth, the cells produced by regular reproduction of nonstem tumor cells. Both the first and second terms use classical logistic growth (note that the carrying capacities for *S* and *T* are distinct). The third term on the RHS represents tumor elimination by CTL in proportion to both *T* and *C*. CTLs are white blood cells responsible for attacking tumor cells, in this case. The third term also introduces the effects of MHC class I receptors (*M*_I_) and TGF-*β* (*F*_*β*_), which is assumed to be a major immunosuppressive factor for CTL activity [10]. Consider

(3) $$\begin{array}{c} S^{\prime }=R_{2}\left(\;S\;\right)S-\alpha \left(\;T,S\;\right)-f_{S}\left(\;F_{\beta }\;\right)g_{S}\left(\;M_{I}\;\right)h_{S}\left(\;S\;\right)CS. \end{array}$$

The first term on the RHS stands for the rate of stem cell growth without immune intervention. As before, this follows a logistical growth model with a carrying capacity, the maximal tumor cell burden. The second term, like the first of the previous equation, stands for differentiated tumor cells produced by CSCs. The third term is almost identical to the third term of the previous equation, except that the functions *f*_*S*_ and *g*_*S*_ represent the interaction of CSCs and CTLs with regard to TGF-*β* and MHC class I (as opposed to TCs in the above equation) [11]. Consider

(4) $$\begin{array}{c} C^{\prime }=f_{C}\left(\;\left(\;T+S\;\right)\cdot M_{II}\;\right)g_{C}\left(\;F_{\beta }\;\right)-\mu_{C}C+N\left(\;t\;\right). \end{array}$$

The first summand of the RHS stands for CTL recruitment from the blood system. The recruitment function is positively affected by MHC class II (*M*_II_) and the number of TCs (*T*) and CSCs (*S*). The cytokine TGF-*β* suppresses the proliferation and activation of T-lymphocytes [2], as well as leukocyte migration across the brain-blood boundary (BBB) [15]; these are collectively represented by the function *g*_*C*_. We assume a constant death rate for *C*, represented by *μ*_*C*_. The term *N*(*t*) describes the rate of infusion of primed CTLs directly to the tumor site. *N*(*t*) is set equal to 0 in absence of immunotherapy [14, 16]. Consider

(5) Fβ′=fβT+S−μβFβ,Fγ′=fγC−μγFγ.

The above two equations describe the dynamics of TGF-*β* and IFN-*γ*, respectively. In the first equation, the first term represents the natural basal level in the CNS (central nervous system). This includes TGF-*β* produced by the tumor, which is assumed to be proportional to the tumor's size. The degradation of TGF-*β* is assumed to be constant with the rate *μ*_*β*_ and is represented by the second term.

In the second equation, the first term on the RHS is a linear production of IFN-*γ*, *F*_*γ*_. We assume the only source of IFN-*γ* is CTL. The second term is the natural degradation of IFN-*γ* with constant rate *μ*_*γ*_ [15]. Consider

(6) MI′=fMIFγ−μMIMI,MII′=fMIIFβgMIIFγ−μMIIMII.

The above two equations represent the dynamics of MHC classes I and II, respectively. For the first equation, the first term on the RHS is the basal rate of *M*_I_ receptor expression per tumor cell. This includes the stimulation by IFN-*γ* of *M*_I_ expression on the surface of a glioblastoma cell. The second term is the natural degradation of *M*_I_ with constant rate *μ*_*M*_I__.

In the second equation, the first term represents the rate of *M*_II_ per tumor cell as a function of IFN-*γ* and TGF-*β* [17]. The second term is the degradation of *M*_II_ with constant rate *μ*_*M*_II__ [15].

## 3. Preliminary Results

Throughout the paper, we will assume that system (1) is subject to nonnegative initial conditions. In addition, the functions *α*, *R*_1_, *R*_2_, *f*_*T*_, *f*_*S*_, *f*_*C*_, *f*_*β*_, *f*_*γ*_, *f*_*M*_I__, *f*_*M*_II__, *g*_*T*_, *g*_*S*_, *g*_*C*_, *g*_*M*_II__, *h*_*T*_, and *h*_*S*_ are all *𝒞* ^1^ functions with nonnegative values. Here, we use *𝒞* ^1^ to denote the space of continuously differentiable functions. These assumptions imply that the nonnegative orthant is invariant under (1) and there exists a unique solution to (1) subject to initial conditions. To ensure solutions to (1) stay bounded over time, we need additional assumptions. We make the following mathematical assumptions modified from [13] to account for cancer stem cells (A1):

(1) *R*_1_(*T*) and *R*_2_(*S*) are at most linear;
(2) *α*(*T*, *S*) is increasing;
(3) *f*_*T*_(*F*_*β*_) and *f*_*S*_(*F*_*β*_) are decreasing and bounded below; *g*_*T*_(*M*_I_) and *g*_*S*_(*M*_I_) are increasing and bounded above;
(4) *h*_*T*_ and *h*_*S*_ are decreasing and bounded below;
(5) *f*_*C*_((*T* + *S*) · *M*_II_) is increasing and bounded above; *g*_*C*_(*F*_*β*_) is decreasing and bounded below;
(6) *N*(*t*) is nonnegative and bounded above;
(7) *f*_*β*_(*T*), *f*_*β*_(*S*), and *f*_*γ*_(*C*) are increasing;
(8) *f*_*M*_I__(*F*_*γ*_) is increasing and bounded above;
(9) *g*_*M*_II__(*F*_*γ*_) is increasing and bounded above; *f*_*M*_II__(*F*_*β*_) is decreasing and bounded below.

We will use the following substitutions to simplify our equations:

(7) Fβ=x,fβ=fx,μβ=μxFγ=y,fγ=fy,μγ=μyMI=u,fMI=fu,μMI=μuMII=v,fMII=fv,μMII=μv.

These substitutions give us the following system equivalent to (1):

(8) T′=αT,S+R1TT−fTxgTuhTTCT,S′=R2SS−αT,S−fSxgSuhSSCS,C′=fCT+S·vgCx−μCC+Nt,x′=fxT+S−μxx,y′=fyC−μyy,u′=fuy−μuu,v′=fvxgvy−μvv.

Also as in [13] and included here for the reader's benefit, we make the following biological assumptions on our system (A2):

(1) *R*_1_(*T*) and *R*_2_(*S*) are decreasing on [0, *K*_1_], [0, *K*_2_], respectively, where *K*_1_ and *K*_2_ are their respective carrying capacities. Furthermore, *R*_1_(*K*_1_) = 0 and *R*_2_(*K*_2_) = 0. Also, *R*_1_(0) = *r*_1_ > 0 and *R*_2_(0) = *r*_2_ > 0.
(2) *α*(*T*, *S*) = *r*_*α*_(*S*/*K*_2_)(*T*/*K*_1_)(*K*_1_ − *T*) (when the CSC population is small, CSCs repopulate themselves, but as their population grows, they focus on populating the TC population) [11].
(3) *f*_*T*_(*x*) and *f*_*S*_(*x*) are decreasing, *f*_*T*_(0) = *f*_*S*_(0) = 1, and lim_*x*→*∞*_ *f*_*T*_(*x*) = *a*_*T*,*x*_, lim_*x*→*∞*_ *f*_*S*_(*x*) = *a*_*S*,*x*_, for some *a*_*T*,*x*_, *a*_*S*,*x*_ > 0 (TGF-*β* decreases the efficacy of tumors killed by CTLs up to some limit).
(4) *g*_*T*_(*u*), *g*_*S*_(*u*) are increasing, *g*_*T*_(0) = *g*_*S*_(0) = 0, and lim_*u*→*∞*_ *g*_*T*_(*u*) = *a*_*T*_ > 0, lim_*u*→*∞*_ *f*_*S*_(*u*) = *a*_*S*_ > 0 (MHC class I receptors are necessary for the action of CTLs and increase their efficiency up to some limit).
(5) *g*_*S*_(*u*) < *g*_*T*_(*u*) (CTLs are less efficient when attacking CSCs) [11].
(6) *h*_*T*_(*T*) and *h*_*S*_(*S*) are decreasing, *h*_*T*_(0) = *h*_*S*_(0) = 1, and lim_*T*→*∞*_ *h*_*T*_(*T*) = 0, lim_*S*→*∞*_ *h*_*S*_(*S*) = 0 (large tumor mass hampers the access of CTLs to tumor cells and reduces their kill rate).
(7) *f*_*C*_(*Tv* + *Sv*) is increasing from 0 to *a*_*C*,*v*_ > 0, *f*_*C*_′(0) > 0, and lim_*Tv*+*Sv*→*∞*_ *f*_*C*_′(*Tv* + *Sv*) = 0 (the total number of MHC class II receptors on all tumor cells and stem cells determines the recruitment of CTLs; the rate of CTL recruitment is limited and its growth decreases to zero).
(8) *g*_*C*_(*x*) is decreasing from 1 to some bound greater than 0 (TGF-*β* reduces recruitment of CTLs).
(9) *f*_*x*_(*T* + *S*) = *g*_*x*_ + *a*_*x*,*T*_ *T* + *a*_*x*,*S*_ *S*, *f*_*y*_(*C*) = *a*_*y*,*C*_ *C*, where *a*_*x*,*T*_, *a*_*x*,*S*_, *a*_*y*,*C*_ > 0 (TGF-*β* and IFN-*γ* are secreted by the cancerous cells and CTLs, resp., at constant rate; there is base level secretion of TGF-*β*).
(10) *f*_*u*_(0) = *g*_*u*_ > 0 and lim_*y*→*∞*_ *f*_*u*_(*y*) = *g*_*u*_ + *a*_*u*,*y*_, *a*_*u*,*y*_ > 0 (there is a constant basic production of MHC class II receptors at the cell surface, while IFN-*γ* increases this production up to some level).
(11) *f*_*v*_(0) = 1 and *f*_*v*_(*x*) is decreasing to 0 (TGF-*β* decreases MHC class II production to 0).
(12) *g*_*v*_(0) = 0, *g*_*v*_(*y*) is increasing to *a*_*u*,*y*_ > 0, *g*_*v*_′ > 0, and lim_*y*→*∞*_ *g*_*v*_′(*y*) = 0 (IFN-*γ* is necessary to induce production of MHC class II receptors and increases up to some level with increase declining to zero).
(13) *N*(*t*) ≡ *N* (treatment is assumed to be constant with respect to time).

**Theorem 1 .**

Under the (A2) assumptions, system (1) is dissipative on [0, *K*_1_]×[0, *K*_2_]×(*ℝ*_≥0_)^5^.

**Proof**

Let *c*_max_ = max{*f*_*C*_(*Tv* + *Sv*)*g*_*C*_(*x*)}, ${\overset{-}{f}}_{x}=(g_{T}+g_{S})+(a_{x,T}K_{1}+a_{x,S}K_{2})\geq f_{x}(T+S)$ for all values of *T* and *S*, ${\overset{-}{f}}_{y}=a_{y,C}C_{max}$, where *C*_max_ = max{(*c*_max_ + *N*)/*μ*_*C*_, *C*(0)}, ${\overset{-}{f}}_{u}=g_{u}+a_{u,y}$, and ${\overset{-}{f}}_{v}=a_{u,y}.$ These values are well defined based on the (A2) assumptions.

In order to show that our system is dissipative, we need to construct a *W* such that

(9) $$\begin{array}{c} \nabla W\cdot F\leq A-\delta W,\;\text{where }A,\delta >0, \end{array}$$

where *F* is the RHS of system (1). Let *W*(*T*, *S*, *C*, *x*, *y*, *u*, *v*) = *T* + *S* + *C* + *x* + *y* + *u* + *v*. Then, since *R*_1_(0) = 0 and *R*_1_(*K*_1_) = 0, *R*_1_(*T*)*T* ≤ *a*_1_ − *b*_1_ *T* for some *a*_1_, *b*_1_ > 0 on [0, *K*_1_]. Similarly, *R*_2_(*S*)*S* ≤ *a*_2_ − *b*_2_ *S* for some *a*_2_, *b*_2_ > 0 on [0, *K*_2_]. Thus,

(10) ∇W·FR1TT−fTxgTuCThTT+R2SS−fSxgSuCShSS+fCTv+SvgCx−μCC+N+fxT+S−μxx+fyC−μyy+fuy−μuu+fvxgvy−μvv≤a1−b1T+a2−b2S+cmax−μCC+N+f−x−μxx+f−y−μyy+f−u−μuu+f−v−μv≤A−δW,

where *δ* = min{*b*_1_, *b*_2_, *μ*_*C*_, *μ*_*x*_, *μ*_*y*_, *μ*_*u*_, *μ*_*v*_} and $A=a_{1}+a_{2}+c_{max}+N+{\overset{-}{f}}_{x}+{\overset{-}{f}}_{y}+{\overset{-}{f}}_{u}+{\overset{-}{f}}_{v}.$

Since our inequalities *R*_1_(*T*)*T* ≤ *a*_1_ − *b*_1_ *T*, *R*_2_(*S*)*S* ≤ *a*_2_ − *b*_2_ *S* hold for all values of *T*, *S* in this space, the system is dissipative everywhere on [0, *K*_1_]×[0, *K*_2_]×(*ℝ*_≥0_)^5^, and by a theorem from Robinson [18], we get the following corollary.

**Corollary 2 .**

System (1) has a compact global attractor on [0, *K*_1_]×[0, *K*_2_]×(*ℝ*_≥0_)^5^.

## 4. Stability Analysis

In the following section, we present an analysis of three potential steady states: tumor elimination (where CSC and TC populations are eradicated), recurrence (where the TC population is eradicated, but the CSC population persists), and coexistence (where CSC and TC populations persist). In each case, we discuss sufficiency conditions on the treatment term *N* which will allow for a globally asymptotically stable cure state (tumor elimination).

### 4.1. Semitrivial Tumor Elimination

We begin our analysis with tumor elimination. Setting *T* = 0 and *S* = 0, we find the equilibrium values:

(11) C∗=NμC,x∗=fx0μx,y∗=fyC∗μy,u∗=fuy∗μu,v∗=fvx∗gvy∗μv.

Substituting this equilibrium point into the Jacobian matrix
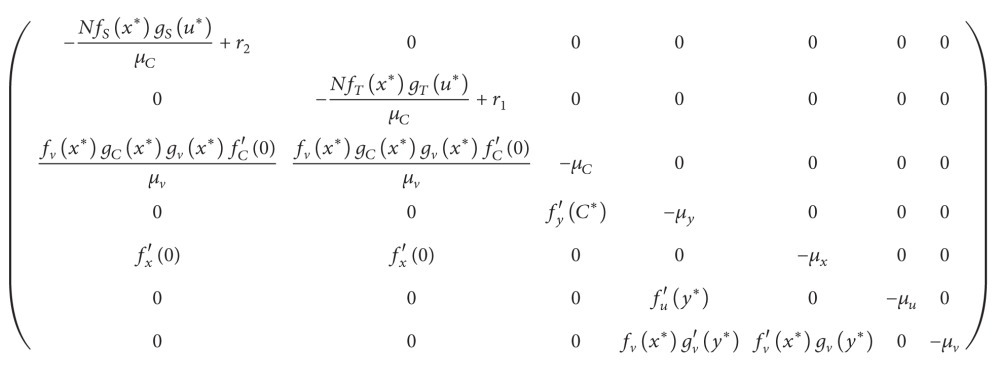
(12)yields eigenvalues *λ* = −*μ*_*C*_, −*μ*_*u*_, −*μ*_*v*_, −*μ*_*x*_, −*μ*_*y*_, −*Nf*_*S*_(*x* ^*∗*^)*g*_*S*_(*u* ^*∗*^)/*μ*_*C*_ + *r*_2_, and −*Nf*_*T*_(*x* ^*∗*^)*g*_*T*_(*u* ^*∗*^)/*μ*_*C*_ + *r*_1_. So as long as we choose *N* large enough so that

(13) N>r2·μCfSx∗gSu∗,N>r1·μCfTx∗gTu∗,

we are guaranteed to have a locally asymptotically stable cure state.

We now show that, under necessary condition (13), (0,0, *C* ^*∗*^, *x* ^*∗*^, *y* ^*∗*^, *u* ^*∗*^, *v* ^*∗*^) is locally asymptotically stable. Let *N* = *N* ^*∗*^, where *N* ^*∗*^ meets condition (13). Then, for some initial conditions, there exists *t*_*ϵ*_ such that, for *t* ≥ *t*_*ϵ*_, *C* > *C* ^*∗*^ = (*N* ^*∗*^/*μ*_*C*_)(1 − *ϵ*) for arbitrarily small *ϵ*. If we also let *y* ^*∗*^ = (*f*_*y*_(*C* ^*∗*^)/*μ*_*y*_)(1 − *ϵ*_1_) and *u* ^*∗*^ = (*f*_*u*_(*y* ^*∗*^)/*μ*_*u*_)(1 − *ϵ*_2_), for *ϵ*_1_, *ϵ*_2_ arbitrarily small, we have that *u* > *u* ^*∗*^ from some starting moment, and therefore *g*_*T*_(*u*)*C* > *g*_*T*_(*u* ^*∗*^)*C* ^*∗*^. By our assumptions (A1), *h*_*T*_(*T*) ≥ *h*_*T*_(*K*_1_) and *f*_*T*_(*x*) ≥ *f*_*T*_(*x*_max_), so if we let *N* ^*∗*^ be large enough so that *g*_*T*_(*u* ^*∗*^)*C* ^*∗*^ > (*r*_1_ + *r*_*α*_ *K*_1_^2^/4)/*h*_*T*_(*K*_1_)*f*_*T*_(*x*_max_), then we have that

(14) T′≤−a1T,where  a1=gTu∗C∗−r1+rαK12/4hTK1fTxmax>0.

A parallel argument works to show that, for *N* ^*∗*^ large enough,

(15) S′≤−a2S,where  a2=gSu∗C∗−r2hSK2fSxmax>0.

Thus, by increasing the treatment value *N*, we are able to show that, for some set of initial conditions, the CSC population *S* and TC population *T* decay exponentially to 0, leading to tumor elimination.

### 4.2. Persistence of Tumor

We now wish to consider steady states in which some subset of cancer cell populations persist. Let our hypothetical equilibrium point be (*T*_*p*_, *S*_*p*_, *C*_*p*_, *x*_*p*_, *y*_*p*_, *u*_*p*_, *v*_*p*_), where *S*_*p*_ > 0. Then

(16) xp=fxTp+Spμx,yp=fyCpμy,up=fuypμu,vp=fvxpgvypμv.

We wish to show existence of *T*_*p*_, *S*_*p*_ > 0, and *C*_*p*_ > 0. To do so, we must solve the system

(17) R1Tp+αTp,Sp=fTfxTp+Spμx·gTfufyCp/μyμuhTTpCp,R2SpSp=αTp,SpTp+fSfxTp+Spμx·gSfufyCp/μyμuhSSpSpCp,μCCp=fCfvfxTp+Sp/μxgvfyCp/μyTp+Spμv·gCfxTp+Spμx+N.

Defining the auxiliary function

(18) HT,SC=fCfvfxT+S/μxgvfyC/μyT+Sμv·gCfxT+Sμx−μCC+N,

we see that, for every *T*, *S* ≥ 0, *H*_*T*,*S*_(0) = *N* > 0.

In addition, taking the derivative with respect to *C*, we get

(19) HT,S′C=fC′fvfxT+S/μxgvfyC/μyT+Sμv·fvfxT+S/μxT+Sμvgv′fyCμyfy′Cμy·gCfxT+Sμx−μC.

From assumptions (A2), we have that *H*_*T*,*S*_′(*C*) is decreasing, so there is exactly one positive *C* for which *H*_*T*,*S*_(*C*) = 0 for any given *T*, *S*. Thus, such *C*_*p*_ > 0 exists, but to further solve system (17), we need more information about the arbitrary functions present.

To move forward in the analysis of system (8), we will need to make the following simplifying assumptions (A3):

(1) The dynamics of TGF-*β* are much faster than those of the other system components.
(2) The inflow of CTLs is constant.

With these assumptions, we can assume *x* is determined by its steady-state *x* ^*∗*^ = *f*_*x*_(*T* + *S*)/*μ*_*x*_ and we get the simplified system:

(20) T′=αT,S+R1TT−f¯TT,SgTuhTTTC,S′=R2SS−αT,S−f¯ST,SgSuhSSSC,C′=−μCC+N,y′=fyC−μyy,u′=fuy−μuu,v′=f¯vT,Sgvy−μvv,

where

(21) f¯TT,S=fTfxT+Sμx,f¯ST,S=fSfxT+Sμx,g¯CT,S=gCfxT+Sμx,f¯vT,S=fvfxT+Sμx.

Note that if *T* = 0, these equations will simply be referred to in terms of *S* and vice versa.

With simplifying assumptions (A3), we are able to study the possible dynamics of persistence of cancer, recurrence, and coexistence, in the following subsections.

### 4.3. Recurrence State Stability

We know that, for system (20), the equilibrium points for *C*, *y*, and *u* must be *C* ^*∗*^ = *N*/*μ*_*C*_, *y* ^*∗*^ = *f*_*y*_(*C* ^*∗*^)/*μ*_*y*_, and *u* ^*∗*^ = *f*_*u*_(*y* ^*∗*^)/*μ*_*u*_. In this section, we wish to study the recurrence steady state, so we will set the TC population *T* = 0 and observe the consequences for the CSC population steady state:

(22) $$\begin{array}{c} S^{\prime }=R_{2}\left(\;S^{*}\;\right)S^{*}-{\bar{f}}_{S}\left(\;S^{*}\;\right)g_{S}\left(\;u^{*}\;\right)h_{S}\left(\;S^{*}\;\right)S^{*}C^{*}=0. \end{array}$$

*S* ^*∗*^ will be a steady state when *S* ^*∗*^ = 0 or

(23) $$\begin{array}{c} R_{2}\left(\;S^{*}\;\right)-{\bar{f}}_{S}\left(\;S^{*}\;\right)g_{S}\left(\;u^{*}\;\right)h_{S}\left(\;S^{*}\;\right)C^{*}=0. \end{array}$$

For further analysis, we denote

(24) GN=gSu∗NC∗N,HS=f¯SShSS,

where *u* ^*∗*^(*N*) = *f*_*u*_(*N*)/*μ*_*u*_. Note that (23) now becomes

(25) $$\begin{array}{c} R_{2}\left(\;S^{*}\;\right)-G\left(\;N\;\right)H\left(\;S^{*}\;\right)=0, \end{array}$$

where *G*(*N*) is increasing in *N* at least linearly and *R*_2_(*S*) and *H*(*S*) are both decreasing. We recall from assumptions (A2) that *R*_2_(*K*_2_) = 0 and $H(K_{2})={\bar{f}}_{S}(K_{2})h_{S}(K_{2})>0$. We define

(26) $$\begin{array}{c} L_{N}\left(\;S\;\right)=R_{2}\left(\;S\;\right)-G\left(\;N\;\right)H\left(\;S\;\right), \end{array}$$

for which we know *L*_*N*_(*K*_2_) < 0 for any *N* > 0.

We also know that *L*_*N*_(0) = *R*_2_(0) − *G*(*N*)*f*_*S*_(*g*_*x*_/*μ*_*x*_). Since *G*(*N*) is an increasing function, its inverse exists and we can define *N*_min_ = *G* ^−1^(*R*_2_(0)/*f*_*S*_(*g*_*x*_/*μ*_*x*_)). Notice that, for *N* = *N*_min_, *L*_*N*_(0) = 0. For *N* < *N*_min_, *L*_*N*_(0) > 0, and *L*_*N*_(*K*_2_) < 0. Therefore, for *N* < *N*_min_, *L*_*N*_(*S*) = 0 has at least one solution *S* ^*∗*^ ∈ (0, *K*_2_) and, in general, since the function must cross the *S*-axis an odd number of times, there are an odd number of solutions, *S*_1_^*∗*^,…, *S*_*n*_^*∗*^.

Without loss of generality, we can assume *C*, *y*, and *u* are at their respective steady states since these variables will all converge to their steady-state values exponentially. We can also assume that, for large enough *t*, (20) is arbitrarily well approximated by

(27) $$\begin{array}{c} S^{\prime }=R_{2}\left(\;S\;\right)S-H\left(\;S\;\right)G\left(\;N\;\right)S=SL_{N}\left(\;S\;\right). \end{array}$$

For *N* < *N*_min_, the equilibrium point (0,0, *C* ^*∗*^, *y* ^*∗*^, *u* ^*∗*^, *v* ^*∗*^) is unstable, since any values of *S* less than 0 will yield a negative value for *L*_*N*_(*S*), and any values of *S* between 0 and *S*_1_^*∗*^ will yield a positive value for *L*_*N*_(*S*). Thus our first stable equilibrium point is at (0, *S*_1_^*∗*^, *C* ^*∗*^, *y* ^*∗*^, *u* ^*∗*^, *v* ^*∗*^).

In contrast, if *N* is large enough so that *N* > *N*_min_, our equilibrium point (0,0, *C* ^*∗*^, *y* ^*∗*^, *u* ^*∗*^, *v* ^*∗*^) is locally stable, since, for *N* > *N*_min_, *L*_*N*_(0) < 0. There could, however, still exist positive solutions *S*_1_^*∗*^,…, *S*_*n*_^*∗*^, where *n* is even. If these solutions are organized in nondecreasing order, *S*_*i*_^*∗*^ is locally stable for even *i* and unstable for odd *i*, since *L*_*N*_(*S*) > 0 between an odd and even root and *L*_*N*_(*S*) < 0 between an even and odd root. Note that if (0,0, *C* ^*∗*^, *y* ^*∗*^, *u* ^*∗*^, *v* ^*∗*^) is the only equilibrium point, it is globally asymptotically stable.

We now show that if we increase our treatment term *N*, we can guarantee the existence of a globally asymptotically stable cure state. Let *ℵ* be the maximum of *R*_2_(*S*)/*G*(*N*_min_)*H*(*S*) and choose *N*_thr_ such that *G*(*N*_thr_) = *G*(*N*_min_)*ℵ*. Notice that this is possible since *G*(*N*) is increasing and continuous. Then for *N* > *N*_thr_, *L*_*N*_(*S*) < 0 for all 0 ≤ *S* ≤ *K*_2_, and so (0,0, *C* ^*∗*^, *y* ^*∗*^, *u* ^*∗*^, *v* ^*∗*^) is now a globally asymptotically stable equilibrium point.

### 4.4. Coexistence State Stability

Still working under simplifying assumptions (A3), we conclude our stability analysis with consideration of a coexistence steady state. As above, for large values of *t*, we can expect *C*, *y*, and *u* to be at steady state, and so we can reduce our system to the two equations

(28) T′R1TT+αT,S−H1T,SG1NT=TL1T,S+rαSK2K1K1−T≡TM1T,S,S′R2SS−αT,S−H2T,SG2NS=SL2T,S−rαTK2K1K1−T≡SM2T,S,

where *G*_1_(*N*) = *g*_*T*_(*u* ^*∗*^(*N*))*C* ^*∗*^(*N*), $H_{1}(T,S)={\bar{f}}_{T}(T,S)h_{T}(T)$, *G*_2_(*N*) = *g*_*S*_(*u* ^*∗*^(*N*))*C* ^*∗*^(*N*), and $H_{2}(T,S)={\bar{f}}_{S}(T,S)h_{S}(S)$. Define *N*_min_ = min⁡{*N*_min,*T*_, *N*_min,*S*_}, where *N*_min,*T*_ = *G*_1_^−1^(*R*_1_(0)/*f*_*T*_(*g*_*x*_/*μ*_*x*_)) and *N*_min,*S*_ = *G*_2_^−1^((*R*_2_(0) − *r*_*α*_ *K*_1_/4*K*_2_)/*f*_*S*_(*g*_*x*_/*μ*_*x*_)).

**Proposition 3 .**

For *N* < *N*_min_, system (28) has a locally stable coexistence steady state.

**Proof**

We begin by showing the existence of such a steady state. Without loss of generality, suppose *N*_min_ = *N*_min,*S*_. Then for values of *N* < *N*_min_, *M*_2_(*T*, 0) > 0 and *M*_2_(*T*, *K*_2_) < 0 for all values of *T* ∈ (0, *K*_1_). Therefore, there exists a value *S* ^*∗*^ ∈ (0, *K*_2_) such that *M*_2_(*T*, *S* ^*∗*^) = 0 for any *T* ∈ (0, *K*_1_). In general, there exist an odd number of solutions *S*_1_^*∗*^,…, *S*_*n*_^*∗*^ such that *M*_2_(*T*, *S*_*i*_^*∗*^) = 0 for any *T* ∈ (0, *K*_1_), *i* = 1,…, *n*.

Likewise, by our choice of *N*_min_, *M*_1_(0, *S*) > 0 and *M*_1_(*K*_1_, *S*) < 0 for all values of *S* ∈ (0, *K*_2_). Therefore, for *N* < *N*_min_, there exists a value *T* ^*∗*^ ∈ (0, *K*_1_) such that *M*_1_(*T* ^*∗*^, *S*) = 0 for all *S* ∈ (0, *K*_2_) and, in general, there exist an odd number of solutions *T*_1_^*∗*^,…, *T*_*m*_^*∗*^ such that *M*_1_(*T*_*j*_^*∗*^, *S*) = 0 for any *S* ∈ (0, *K*_2_), *j* = 1,…, *m*.

Thus, we are able to deduce the existence of an equilibrium point (*T* ^*∗*^, *S* ^*∗*^) in (0, *K*_1_)×(0, *K*_2_) for system (28). Moreover, notice that while (0,0) is an equilibrium point of system (28), it is unstable since *M*_1_(*T*, *S*) > 0 for 0 ≤ *T* < *T*_1_^*∗*^, *S* ∈ (0, *K*_2_), and *M*_2_(*T*, *S*) > 0 for 0 ≤ *S* < *S*_1_^*∗*^, *T* ∈ (0, *K*_1_). In fact, if we denote *T*_0_ = 0 and *S*_0_ = 0, equilibrium points (*T*_*j*_^*∗*^, *S*_*i*_^*∗*^), *j* = 0,…, *m*, *i* = 0,…, *n*, are locally unstable when *i* or *j* is even and locally stable when *i* and *j* are odd.

**Remark 4 .**

Note that, in the absence of treatment, *N* = 0 < *N*_min_. Therefore, in the case where the tumor is left untreated, cancer persists.

We now show that, by increasing the treatment term *N*, we will achieve a globally asymptotically stable cure steady state. For *N* ≥ *N*_min_, let us consider the system

(29) T′=TL1T,S+rαK1,S′=SL2T,S.

Define *ℵ*_*S*_ as the maximum of *R*_2_(*S*)/*G*_2_(*N*_min,*S*_)*H*_2_(*K*_1_, *S*) and choose *N*_thr,*S*_ such that *G*_2_(*N*_thr,*S*_) = *G*_2_(*N*_min,*S*_)*ℵ*_*S*_. Similarly, let *ℵ*_*T*_ be the maximum of (*R*_1_(*T*) + *r*_*α*_ *K*_1_)/*G*_1_(*N*_min,*T*_)*H*_1_(*T*, *K*_2_) and choose *N*_thr,*T*_ such that *G*_1_(*N*_thr,*T*_) = *G*_1_(*N*_min,*T*_)*ℵ*_*T*_. Let *N*_cure_ = max{*N*_thr,*T*_, *N*_thr,*S*_}.

**Proposition 5 .**

For *N* > *N*_*cure*_, (0,0) is a globally asymptotically stable equilibrium point of system (29).

**Proof**

For *N* > *N*_thr,*T*_,

(30) L1T,S+rαK1R1T−G1Nthr,TH1T,S+rαK1=R1T−ℵTG1Nmin,TH1T,S+rαK1=R1T+rαK1−H1T,SH1T,K1R1T+rαK1≤0

for 0 ≤ *T* ≤ *K*_1_ since *H*_1_ is decreasing based on assumptions (A2). Thus, *L*_1_(*T*, *S*) + *r*_*α*_ *K*_1_ < 0 for all (*T*, *S*)∈[0, *K*_1_]×[0, *K*_2_]. Similarly, for *N* > *N*_thr,*S*_, *L*_2_(*T*, *S*) < 0 for all (*T*, *S*)∈[0, *K*_1_]×[0, *K*_2_]. Therefore, (0,0) is the only equilibrium point for system (29) and (0,0) is globally asymptotically stable.

We conclude our analysis by noting that

(31) T′TL1T,S+rαSK2K1K1−T≤TL1T,S+rαK1S′SL2T,S−rαTK2K1−T≤SL2T,S

for all (*T*, *S*)∈[0, *K*_1_]×[0, *K*_2_].

**Corollary 6 .**

For *N* > *N*_*cure*_, (0,0) is a globally asymptotically stable equilibrium solution to system (28).

## 5. Example

In this section, we present an example to illustrate the theory presented above. This model is a modification of the biologically verified system presented in Kronik et al. [10] to address the CSC Hypothesis as presented in this paper. Modifications include the incorporation of CSCs and amending the CTL equation to satisfy assumptions (A3). Consider the following system:

(32) dTdt=r1T1−TK1+rαTK1SK2K1−T−aTMIMI+eTaT,β+eT,β1−aT,βFβ+eT,βCThT+T,dSdt=r2S1−SK2−rαTK1SK2K1−T−aSMIMI+eSaS,β+eS,β1−aS,βFβ+eS,βCShS+S,dCdt=−μCC+N,dFβdt=gβ+aβ,TT+aβ,SS−μβFβ,dFγdt=aγ,CC−μγFγ,dMIdt=gMI+aMI,γFγFγ+eMI,γ−μMIMI,dMIIdt=aMII,γFγFγ+eMII,γeMII,β1−aMII,βFβ+eMII,β+aMII,β−μMIIMII

subject to the initial conditions *T*(0) = 70, *S*(0) = 30, *C*(0) = 250, *F*_*β*_(0) = 50, *F*_*γ*_(0) = 50, *M*_I_(0) = 50, and *M*_II_(0) = 50. Parameter values are given by Table 1; calculations for these parameter values can be found in [8, 10, 20].

The equilibrium values for *C*, *F*_*β*_, *F*_*γ*_, *M*_I_, and *M*_II_ are

(33) C∗=NμC,Fβ∗=gβ+aβ,SS+aβ,TTμβ,Fγ∗=aγ,CC∗μγ,MI∗=eMI,γgMI+aMI,γ+gMIFγ∗μMIeMI,γ+Fγ∗,MII∗=aMII,γeMII,β+aMII,βFβ∗Fγ∗μMIIeMII,β+Fβ∗eMII,γ+Fγ∗.

Following calculations from Section 4.4, we get *G*_1_(*N*) = (*a*_*T*_(*g*_*M*_I__ + *a*_*M*_I_,*γ*_ *F*_*γ*_^*∗*^/(*F*_*γ*_^*∗*^ + *e*_*M*_I_,*γ*_))/(*g*_*M*_I__ + *a*_*M*_I_,*γ*_ *F*_*γ*_^*∗*^/(*F*_*γ*_^*∗*^ + *e*_*M*_I_,*γ*_) + *e*_*T*_))(*N*/*μ*_*C*_) and *G*_2_(*N*) = (*a*_*S*_(*g*_*M*_I__ + *a*_*M*_I_,*γ*_ *F*_*γ*_^*∗*^/(*F*_*γ*_^*∗*^ + *e*_*M*_I_,*γ*_))/(*g*_*M*_I__ + *a*_*M*_I_,*γ*_ *F*_*γ*_^*∗*^/(*F*_*γ*_^*∗*^ + *e*_*M*_I_,*γ*_) + *e*_*S*_))(*N*/*μ*_*C*_). This gives

(34) Nmin,T=G1−1r1aT,β+eT,β1−aT,β/gβ/μβ+eT,β=G1−1.00117=.00245,Nmin,S=G2−1r2−rαK1/4K2aS,β+eS,β1−aS,β/gβ/μβ+eS,β=G2−1.09976=2.07889.

**Figure 1:**
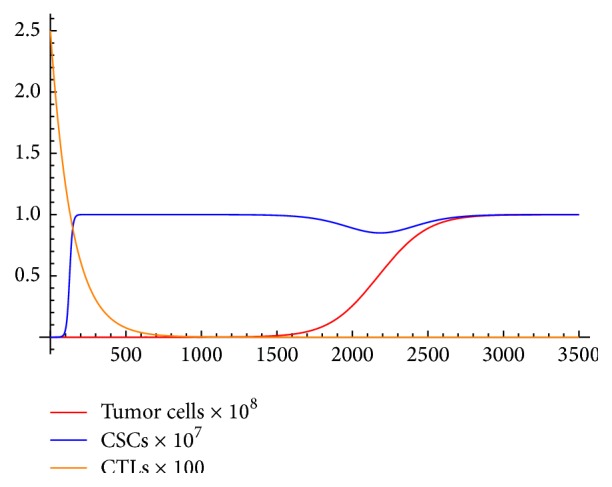
*N* = 0.

Thus, in the case of no treatment, cancer will persist (see Figure 1).

**Figure 2:**
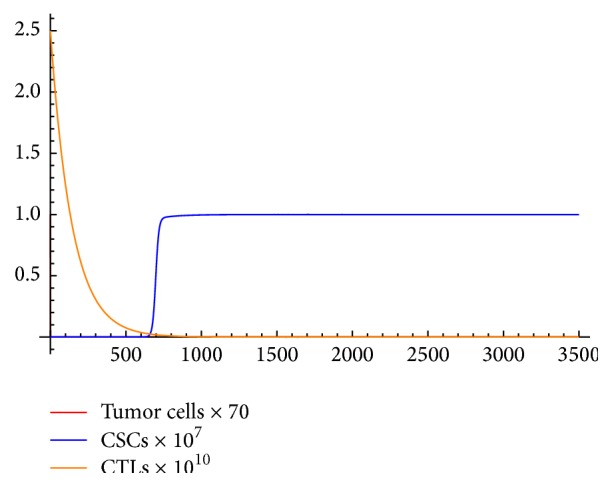
A locally stable recurrence state.

When we increase treatment past *N*_min_, we are able to find initial conditions that will allow a locally stable cure or recurrence state. This can be seen by adding a treatment value of *N* = 1 and increasing our initial amount of CTLs to *C*(0) = 2.5*∗*10^10^ (see Figure 2).

When we maximize (*R*_1_(*T*) + *r*_*α*_ *K*_1_)/*G*_1_(*N*_min,*T*_)*H*_1_(*T*, *K*_2_) on the interval [0, *K*_1_], we calculate *ℵ*_*T*_ = 3.60322*∗*10^17^. Similarly, when we maximize *R*_2_(*S*)/*G*_2_(*N*_min,*S*_)*H*_2_(*T*, *S*) on the interval [0, *K*_2_], we calculate *ℵ*_*S*_ = 1.4866*∗*10^15^. Since *G*_1_(*N*_min,*T*_) = .00245 and *G*_2_(*N*_min,*S*_) = 2.07889, we have

(35) Nthr,TG1−1.00245∗3.60322∗1017=3.10199∗1014,Nthr,SG2−12.07889∗1.4866∗1015=1.08783∗1015.

**Figure 3:**
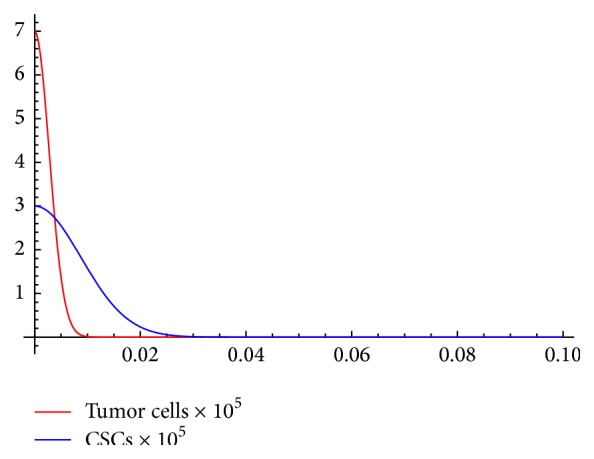
Even with large initial populations of tumor cells *T*(0) = 7*∗*10^5^ and CSCs *S*(0) = 3*∗*10^5^, a cure state is rapidly achieved when *N* = 1.08783*∗*10^15^.

Taking the maximum of these two values, we find *N*_cure_ = 1.08783*∗*10^15^ (see Figure 3).

## 6. Conclusion

In this paper we extend a previous model for the treatment of glioblastoma multiforme with immunotherapy by accounting for the existence of cancer stem cells that can lead to the recurrence of cancer when not treated to completion. We prove existence of a coexistence steady state (one where both tumor and cancer stem cells survive treatment), a recurrence steady state (one where cancer stem cells survive treatment, but tumor cells do not; hence, upon discontinuation of treatment, the tumor would be repopulated by the surviving cancer stem cells), and a cure state (one where both tumor and cancer stem cells are eradicated by treatment). Furthermore, we categorize the stability of the previously mentioned steady states depending on the amount of treatment administered. Finally, in each case, we establish sufficiency conditions on the treatment term for the existence of a globally asymptotically stable cure state. It should be noted that the amount of treatment necessary to eliminate all cancer stem cells as well as tumor cells is higher than the amount of treatment necessary to merely eliminate tumor cells, based on the lower value of *g*_*S*_ (see (A2)). These results are an improvement on previous models that do not account for the existence of cancer stem cells and therefore yield an artificially low value of treatment necessary for a cure state.

However, it is important to note that the values of treatment for which we achieve a globally stable cure state are sufficient, but not necessary. We make the assumption that the production of cytotoxic-T-lymphocytes is constant in order to simplify our analysis (see (A2) and (A3)), leading to a potentially higher-than-necessary value of treatment (since the treatment would ordinarily be helped along by the body's natural production of CTLs, not relying on the treatment, *N*, alone). A natural extension of this work would account for the body's natural production of CTLs in the analysis of the recurrence and coexistence steady states, as well as allowing the treatment term *N* to vary over time.

## Figures and Tables

**Table 1:** Parameter values.

Parameter	Value	Units	Reference
*r*_1_	.001	h^−1^	Based on data from Burger et al. [19]
*K*_1_	10^8^	Cell	Turner et al. [20]
*a*_*T*_	.12	h^−1^	Based on data from Arciero et al. [21] and Wick et al. [22]
*e*_*T*_	50	rec·cell^−1^	Based on data from Kageyama et al. [23]
*a*_*T*,*β*_	.69	None	Thomas and Massagué [2]
*e*_*T*,*β*_	10^4^	pg	Based on data from Peterson et al. [24]
*h*_*T*_	5*∗*10^8^	Cell	Based on data from Kruse et al. [14]
*r*_2_	.1	h^−1^	Vainstein et al. [8]
*K*_2_	10^7^	Cell	Turner et al. [20]
*r*_*α*_	.006	h^−1^	Vainstein et al. [8]
*a*_*S*_	.1*∗a*_*T*_	h^−1^	Estimated based on data from Prince et al. [11]
*e*_*S*_	*e*_*T*_	rec·cell^−1^	Estimated
*a*_*S*,*β*_	*a*_*T*,*β*_	None	Estimated
*e*_*S*,*β*_	*e*_*T*,*β*_	pg	Estimated
*h*_*S*_	*h*_*T*_	Cell	Estimated
*μ*_*C*_	.007	h^−1^	Taylor et al. [25]
*g*_*β*_	6.3945*∗*10^4^	pg·h^−1^	Peterson et al. [24]
*a*_*β*,*T*_	5.75*∗*10^−6^	pg·cell^−1^·h^−1^	Peterson et al. [24]
*a*_*β*,*S*_	*a*_*β*,*T*_	pg·cell^−1^·h^−1^	Estimated
*μ*_*β*_	7	h^−1^	Coffey Jr. et al. [26]
*g*_*M*_I__	1.44	rec·cell^−1^·h^−1^	Based on data from Kageyama et al. [23]
*a*_*M*_I_,*γ*_	2.88	rec·cell^−1^·h^−1^	Based on data from Yang et al. [27]
*e*_*M*_I_,*γ*_	3.38*∗*10^5^	pg	Based on data from Yang et al. [27]
*μ*_*M*_I__	.0144	h^−1^	Milner et al. [28]
*a*_*M*_II_,*γ*_	8660	rec·cell^−1^·h^−1^	Based on data from Phillips et al. [29] and Bosshart and Jarrett [30]
*e*_*M*_II_,*γ*_	1420	pg	Based on data from Phillips et al. [29] and Bosshart and Jarrett [30]
*a*_*M*_II_,*β*_	.012	None	Based on data from Suzumura et al. [17]
*e*_*M*_II_,*β*_	10^5^	pg	Based on data from Suzumura et al. [17]
*μ*_*M*_II__	.0144	h^−1^	Based on data from Lazarski et al. [31]
*a*_*γ*,*C*_	1.02*∗*10^−4^	pg·cell^−1^·h^−1^	Kim et al. [32]
*μ*_*γ*_	.102	h^−1^	Turner et al. [33]
